# *Artemisia pallens* W. Attenuates Inflammation and Oxidative Stress in Freund’s Complete Adjuvant-Induced Rheumatoid Arthritis in Wistar Rats

**DOI:** 10.3390/diseases12100230

**Published:** 2024-09-29

**Authors:** Tasneem Ahmad, Parag Kadam, Gopal Bhiyani, Hasan Ali, Md. Akbar, Mohd Usman Mohd Siddique, Mudassar Shahid

**Affiliations:** 1 School of Pharmacy, Al-Karim University, Katihar 854106, Bihar, India; ahmad.tasneem0786@gmail.com; 2 Poona College of Pharmacy, Bharati Vidyapeeth Deemed University, Erandawane, Pune 411038, Maharashtra, India; parag.kadam24@gmail.com; 3 Department of Pharmacy, Meerut Institute of Technology, Dr. A. P. J. Abdul Kalam Technical University (AKTU), Meerut 250103, Uttar Pradesh, India; gopal.bihani007@gmail.com (G.B.); hasanmesra@gmail.com (H.A.); 4 Department of Pharmaceutical Chemistry, Shri Vile Parle Kelavani Mandal’s Institute of Pharmacy Dhule (MH), Dhule 424001, Maharashtra, India; 5 Department of Pharmaceutics, College of Pharmacy, King Saud University, Riyadh 11451, Saudi Arabia; mahmad1@ksu.edu.sa

**Keywords:** arthritis, inflammation, oxidative stress, cartilage, bone, herbal extract

## Abstract

Rheumatoid arthritis (RA) is an autoimmune disease that causes distinctive inflammatory symptoms and affects over 21 million people worldwide. RA is characterized by severe discomfort, swelling, and degradation of the bone and cartilage, further impairing joint function. The current study investigates the antiarthritic effect of a methanolic extract of *Artemisia pallens* (methanolic extract of *A. pallens*, MEAP), an aromatic herb. Artemisinin content (% per dry weight of the plant) was estimated using a UV Vis spectrophotometer. In the present study, animals were divided into six groups (*n* = 6). The control group (group I) was injected with 0.25% of carboxymethyl cellulose. The arthritic control group (group II) was treated with Freund’s complete adjuvant (by injecting 0.1 mL). Prednisolone (10 mg/kg), a lower dose of MEAP (100 mg/kg), a medium dose of MEAP (200 mg/kg), and a higher dose of MEAP (400 mg/kg) were orally delivered to groups III, IV, V, and VI, respectively. Freund’s complete adjuvant was administered into the sub-plantar portion of the left-hind paw in all the groups except vehicle control to induce rheumatoid arthritis. Weight variation; joint diameter; paw volume; thermal and mechanical hyperalgesia; hematological, biochemical, and oxidative stress parameters; radiology; and a histopathological assessment of the synovial joint were observed in order to evaluate the antiarthritic effect of the methanolic extract of *A. pallens*. In this study, the estimated content of artemisinin was found to be 0.28% (per dry weight of the plant), which was in good agreement with the reported value. MEAP (200 and 400 mg/kg) caused a significant reduction in increased paw volume and joint diameter in arthritic rats while significantly increasing body weight and the mechanical threshold of thermal algesia. Moreover, complete blood counts and serum enzyme levels improved significantly. Radiological analysis showed a reduction in soft tissue swelling and small erosions. A histopathological examination of the cells revealed reduced cell infiltration and the erosion of joint cartilage in MEAP-administered arthritic rats. The present research suggests that the antiarthritic activity of the methanolic extract of *A. pallens* wall is promising, as evidenced by the findings explored in our rat model.

## 1. Introduction

Rheumatoid arthritis (RA) is an autoimmune disease with characteristic inflammatory conditions, affecting over 21 million people across the globe. Females are three times more prone to RA compared to males [1]. Severe pain, swelling, and bone and cartilage erosion impair joint functionality, and the disease is characterized by various other signs (inflamed joints and swelling) and symptoms (fever, morning stiffness, and tenderness) [2]. The strong evidence to support RA being a systemic autoimmune illness includes the detection of autoantibodies (autoAbs) against circulated proteins, immunoglobulins (rheumatoid factor), and other endogenous proteins in the general systemic circulation [3,4,5].

Among several treatment approaches, non-steroidal anti-inflammatory drugs, immunosuppressants, steroids, and disease-modifying anti-rheumatic drugs are commonly recommended treatment strategies for RA treatment [6]. However, the adverse effects of these medications include bronchospasm, renal damage, stomach ulcers, and cardiac irregularities, resulting in limited clinical use [7]. Considering natural products (alkaloids, steroids, polyphenols, coumarins, terpenes, and flavonoids) derived from medicinal plants as safe and effective treatment alternatives has drawn significant attention from researchers, academics, and scientists. This can be rationalized based on their numerous pharmacological benefits (analgesic, anti-inflammatory, and antiarthritic properties, with fewer side effects) [8,9].

**Figure 1:**
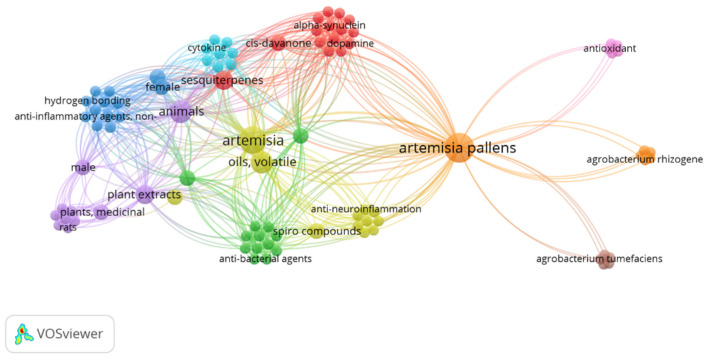
The occurrence of keyword “*Artemesia pallens* wall” in PubMed central with 6 clusters over the period of 1999–2024.

*Artemisia pallens* Wall belongs to the family *Asteraceae*. It is an aromatic plant that grows widely in humid environments throughout Indian plains [10]. It is commercially grown for its aromatic flowers and leaves. In folk medicine, its dried flowering tops are used to cure inflammation and urinary problems [11,12]. Moreover, its oil has stimulant, antispasmodic, antibacterial, and antifungal activities [13]. Furthermore, *A. pallens* is used to treat wounds and diabetes mellitus. Sesquiterpene lactone is a powerful anti-inflammatory molecule that gives *A. pallens* Wall its anti-inflammatory action. Mohammed et al. reported the strong healing and immune-stimulant potential of Artemisia oil for treating second-degree skin burns in a rat model. Notably, this potential healing property was associated with the anti-inflammatory and antioxidant abilities of the extract due to the presence of cinnamate derivatives and oxygenated monoterpenes [14]. Xie et el. reported comprehensive review findings on the use of artemisinin and its derivative as an anti-inflammatory agent to treat autoimmune diseases such as RA, systemic lupus erythematous, immune-mediated kidney disease, osteoarthritis, psoriasis, atopic dermatitis, immune-mediated gastric diseases, multiple sclerosis, autoimmune myasthenia gravis, neuroinflammation, autoimmune thyroiditis, diabetes, immune-mediated exocrine diseases, and related complications [15]. Its herbal formulations are well-known Chinese traditional medicines that have been used to cure severe pain and inflammation for about 100 years [16]. The drug has a wide range of research gaps for further exploration of its multiple functionalities and clinical benefits, as evidenced by the limited research explored so far. The keyword “*Artemesia pallens* wall” in Pubmed Central resulted in only 13 outcomes from 1999 to 2024 (accessed on 6 August 2024). The occurrence of this keyword was visualized using the VOSviewer program (VOSviewer 1.6.20, Leiden University, Netherlands), as shown in Figure 1.

Considering several adverse effects of conventional synthetic drugs, natural *Artemisia pallens* could be a promising alternative to treat RA. Initially, the plant was collected and identified from authentic sources. The content of artemisinin was estimated using a UV vis spectrophotometer technique by constructing a calibration curve (standard artemisinin). The present research study addressed the estimation of the antiarthritic activity of the ethanolic extract of *A. pallens* wall in Freund’s complete adjuvant (FCA)-stimulated arthritis in Wistar rats. The extract was explored for its pharmacological activities and the impact of doses on body weight in a rat model for a period of 28 days. A histopathological examination corroborated its safety concerns explored in the rats. This study is the first to investigate the explored concentrations in a rat model so far.

## 2. Materials and Methods

### 2.1. Chemicals and Reagents

Freund’s complete adjuvant (FCA) was procured from Sigma Aldrich, Mumbai, India. Acurex Biomedical Pvt. Ltd. (Mumbai, Maharashtra, India), provided various biochemical kits as diagnostic kits, such as alanine aminotransferase (ALT, Alkaline Phosphatase 300), aspartate aminotransferase (AST, GOT (AST) 200), and alkaline phosphatase (ALP, AutoPure Alkaline Phosphatase 100 Reagent) (Mumbai, Maharashtra 400016, India). Artemisinin was procured from Sigma Aldrich. Mumbai, India. Water for injection was used as an aqueous medium for injection purposes. Methanol was procured from SD Fine Chemical, Mumbai, India. The saline solution was obtained from a local medical shop. Prednisolone was procured from Merck, Mumbai, India. Formalin and formic acid were purchased from Fisher Scientific Pvt. Ltd., Mumbai, India.

### 2.2. Collection and Identification of the Plant

The aerial portions of *A. pallens* Wall were collected from Jejuri, Pune, Maharashtra, India. Dr. A. S. Upadhye, Department of Botany, Agharkar Research Institute in Pune, India, has identified and verified the aerial sections of *A. pallens* Wall. For future reference, a voucher specimen (Voucher specimen No. WP-091) was deposited at the Agharkar Research Institute in Pune, India.

### 2.3. Extraction Procedure

The maceration method was used to obtain the extract from the coarsely powdered aerial parts of *A. pallens* using methanol. The leaves were dried (in air) for a week at room temperature (25 °C). A total of 100 g of air-dried powder (mesh size of 16#) from *A. pallens* aerial parts was macerated in methanol (1 L) for 24 h at room temperature with occasional shaking. To obtain a dark green extract, the macerate was first filtered through a Whatman filter (No. 1) and the filtrate was evaporated, employing a vacuum evaporator (Rotava, Equitron Instruments) at 50 ± 1 °C following the reported method with a slight modification [17]. The organic solvent was completely removed to obtain the dried form of the extract (22.5 g/L). For further investigation, the methanolic extract of *A. pallens* Wall was coded as MEAP [18].

### 2.4. Analytical Method of Artemisinin Estimation Using UV Vis Spectrophotometer

To estimate the content of artemisinin from the extract, pure artemisinin was first dissolved in methanol to prepare a stock solution. A weighed amount of artemisinin (20 mg) was completely dissolved in methanol (200 mL). A serial dilution was performed to obtain a series of concentrations (20, 40, 60, 80, and 100 µg/mL) for constructing a standard curve. A linear regression curve was constructed using absorbance (520 nm) and corresponding concentration. The mean absorbance value (*n* = 3) was used to construct the curve. The absorbance was estimated using a UV Vis spectrophotometer (U-1800, Shimadzu, Tokyo, Japan). The extract of the plant was prepared in methanol and the extract was filtered through 0.45 µm membrane filter. The extract was purified for artemisinin using silica gel (5 g dry weight). The silica gel was added into a 249 mL volumetric flask containing n-hexane (100 mL). Then, the methanolic extract (1 mL) was added to it. The mixture was allowed to shake (constant agitation) for 5 h to reach the adsorption equilibrium at room temperature. Finally, the saturated silica gel was filtered and washed with hexane 3–4 times until discoloration of the filtrate. The filtrate was then dried over a rotary evaporator. The content of artemisinin (%) from the gel was estimated using the absorbance value at 520 nm and calculated using the slope and intercept of the calibration curve [18].

### 2.5. High-Performance Liquid Chromatography (HPLC) Technique

To ensure an accurate estimation of artemisinin from the extract, the HPLC method was adopted, following the reported method, with slight modifications [19]. Analysis was carried out using the HPLC coupled with a C18 column (an X-Bridge as high-purity base-deactivated silica) of standard dimensions (250 × 4.6 mm, 5 µm of particle size for the packing material). The system column was operated at 30 °C using a photodiode array (PDA) detector (Alliance e2695, Waters Corporation, 34 Maple Street, Milford, MA, USA). The mobile contained methanol and 0.01 M phosphate buffer solution (40:60, *v*/*v*) (at optimal pH 6.6). The mobile phase was degassed using an ultra-sonic water bath for 15 min, followed by membrane filter (0.45 µm). Analysis was conducted using a minimum injection volume (10 µL), flow rate of 0.5 mL/min, and total run time of 15 min. A standard working stock solution (1 mg/mL) of pure artemisinin was freshly prepared in the mobile phase. Various concentrations were prepared using the stock solution after a series of dilutions (0.01–1.0 µg/mL). A linear calibration curve was prepared with high regression coefficient (r^2^ = 0.999). The content of artemisinin from the methanolic extract was estimated using the same experimental operating conditions. The experiment was replicated to obtain a mean and standard deviation.

### 2.6. Experimental Animals

Two animal models were used in the study. Albino mice (weighing about 18–23 g) were 6–7 months old, whereas Wistar rats (~180–210 g) were 1–2 years old. These animals were received from the National Institute of Biosciences, Pune, Maharashtra, India. Animals were placed in the animal house with standard hygienic conditions, which included a 12 h day and night cycle and ambient temperature (20 ± 2 °C) with free access to food and water (air-conditioned room). All animals were issued by the Institutional Animal Ethics Committee (IAEC) after approval. Animals were approved by the College PCTE (PCTE/LDH/IAEC/1370) to conduct the study as per the ARRIVE protocol and OECD guidelines. The study protocol was reviewed and approved by the ethical committee for the research objectives. The committee members approved the study design, protocol (as per the ARRIVE guidelines), the number of animals, and the groups. Moreover, they suggested guiding the blood collection scheme and the maximum limit of blood collection.

### 2.7. Acute Oral Toxicity Study

In compliance with OECD guidelines 425, the acute oral toxicity study was conducted on healthy female Swiss albino mice. After an overnight fast, mice animals were randomly divided into five groups (I, II, III, IV, and V). A single dose of 2000 mg/kg body weight of MEAP was orally administered. The dosed mice were constantly monitored for autonomic and behavioral profiles for 14 days. Initially, each dosed animal was carefully observed at 2, 4, 8, 12, and 48 h to observe any possible changes (immediate effects) within 48 h. Furthermore, animals were inspected for possible signs of toxicity and mortality for up to 14 days [19].

### 2.8. FCA-Induced Arthritis

In the study, Wistar rats (36) were randomly divided into six (*n* = 6) groups. Each rat weighed around 180–210 g. The control vehicle was 0.25% carboxymethyl cellulose suspension (group I). Groups II, III, IV, V, and VI were positive arthritic control, prednisolone (10 mg/kg, p.o.)-treated, low dose of MEAP (100 mg/kg, p.o.)-treated, medium dose of MEAP (200 mg/kg, p.o.)-treated, and high dose of MEAP (400 mg/kg, p.o.)-treated groups, respectively.

On day 0, arthritis was induced by direct injection of FCA (0.1 mL) into the sub-plantar region of the left hind paw. Arthritis was developed in all animals except the vehicle control group. The animals were observed on a daily basis by checking the affected paws, body weight, and general condition of the investigated animals. After a few hours (until 28 days), a sub-plantar injection of FCA in rats resulted in a noticeable local edema that expanded over time and reached the peak on the 12th day following inoculation. Dosing with the test compound began with varied doses and continued until the 28th day [20].

### 2.9. Measurement of Body Weight

Treatment with the extract was expected to cause variation in body weight of the treated groups. Therefore, it was mandatory to observe body weight variation from day 0 to day 28. On days 0, 12, and 28 after FCA injection, body weight was measured using a digital weighing balance (Contech Instruments Co. PCP/B. Pharm/02-03, Navi Mumbai, India). Each animal was individually weighed, and the mean weight was obtained after a repeated weighing process (*n* = 6).

### 2.10. Measurement of Paw Volume

Rats are highly sensitive to the injected compound, particularly the arthritis-inducing agent. Edema and swelling are the result of inflammatory reactions. Therefore, it is a well-established method and diagnostic symptom to confirm induced arthritis. In brief, the volume of the left hind paw was measured on day 0 (before FCA injection) and on days 1, 12, and 28 following FCA injection, employing a plethysmometer. Full edema was developed 5–6 h after injection. As arthritis progressed, redness, swelling, and severe pain in knee joints started to appear. These symptoms maximally appeared on day 1 post-injection. Data have been shown as the volume increases relative to day 0 [21].

### 2.11. Measurement of Joint Diameter

On day 0 (before the FCA injection) and on days 1, 12, and 28 following the adjuvant injection, the joint diameter of each animal was measured. The joint diameter was precisely scaled by a digital Mitutoyo Digimatic Caliper (100 Lauman Lane, Ste A, Hicksville, NY 11801, USA) [4]. The experiment was replicated to report an average value with standard deviation.

### 2.12. Measurement of Mechanical Hyperalgesia

Mechanical hyperalgesia was estimated using the Von Frey Hair apparatus (ALMEMO 2390-5) on day 0 (before the FCA injection) and on days 12 and 28 following the induction of arthritis by FCA, in accordance to the method described by Lee [22].

### 2.13. Measurement of Thermal Hyperalgesia

To inspect the diseased condition of the FCA-treated rats, thermal hyperalgesia was evaluated. Rats were allowed to spend at least ten minutes getting used to the testing environment before behavioral testing began. Once the rat was acclimated to its new environment, the planter surface of its hind paw was heated with the radiant energy until the rodent lifted its paw. When the animal withdrew its paw, the reflected light beam was disrupted, causing a photoelectric cell to automatically switch off the heat. Paw withdrawal latency (PWL) was measured, with a cut-off time of 15 s [23]. Thermal hyperalgesia was measured on day 0 (pre-FCA injection), 12, and 28 after FCA induction of arthritis using a commercially available thermal plantar tester (UGO Basile S.R.L. Via Giuseppe di Vittorio, 2 21036 Gemonio (VA), Italy).

### 2.14. Hematological and Biochemical Parameter Assessment

On the 28th day after blood withdrawal via retro-orbital puncture, hematological parameters such as hemoglobin (Hb), white blood cell (WBC), red blood cell (RBC), and platelets were determined using Sysmax KX-21. Moreover, the prime biochemical biomarkers (ALT, AST, and ALP) were estimated for a comparative assessment against the control (untreated) group [24].

### 2.15. Antioxidant Activity

The rats were sacrificed on the 28th day of therapy by cervical dislocation, and the liver of each animal was taken out followed by immediate rinsing with ice-cold saline. The tissue homogenates were prepared in a tris-HCl buffer (0.1 M) of pH 7.4. Malonidialdehyde (MDA), superoxide dismutase (SOD), and reduced glutathione (GSH) were measured using the supernatant. The GSH and SOD malondialdehyde were estimated as per the reported methods [25,26,27].

### 2.16. Measurement of Spleen Weight

Using the cervical dislocation method, animals were ethically sacrificed on the 28th day. Then, the spleen was excised and weighed. The difference in weight variation was compared against the control group [28].

### 2.17. Radiography

The animals were anesthetized on the last day of treatment, and a CR-30-X machine (AGFA Healthcare N.V. Septestraat 27, Flemish Region and the province of Antwerp, Mortsel, Belgium) was used for radiographic assessment of the hind paws on Fuji AGFA film. In each animal, radiographs were examined for periosteal new bone growth and bone erosion, soft tissue edema, and bone matrix resorption [29].

### 2.18. Estimation of Arthritis Damage

The knee joints were removed on the 28th day after ethical sacrifice and fixed in 10% formaldehyde. A 5 % formic acid solution was used to decalcify tissues, and then it was prepared for paraffin embedding in cube formation. The tissue samples were properly sliced into small pieces with fine thickness (5 µm). The sample was picked up on the glass slide for staining using the hematoxylin (basic) and eosin (acidic) dyes. The stained tissue was visualized under a light microscope to examine the histological changes, if they occurred (hyperplasia of the synovium, formation of the pannus, and deterioration of the joint space) [16,30,31]. In the histopathological staining, the eosin (acidic stain) and hematoxylin (basic dye) stained the cytoplasmic and intracellular organs, respectively, for clear visualization of the changes.

### 2.19. Statistical Analysis

Each experimental analysis and procedure was replicated to obtain a mean and standard error of the mean (SEM) (data expressed as mean ± SD). Graph Pad Prism 5.0 (Graph Pad, San Diego, CA, USA) was utilized to analyze the data. After two-way analysis of variance (ANOVA), the results on body weight, joint diameter, paw volume, thermal hyperalgesia, and mechanical parameters were examined using the Bonferroni post hoc test. Following one-way ANOVA to examine the data on the hemodynamic and biochemical parameters, Dunnett’s test was carried out. A value was considered as significant in statistical analysis if *p* ˂ 0.05.

## 3. Result

### 3.1. Analytical Methodology to Estimate Artemisinin from the Extract

**Figure 2:**
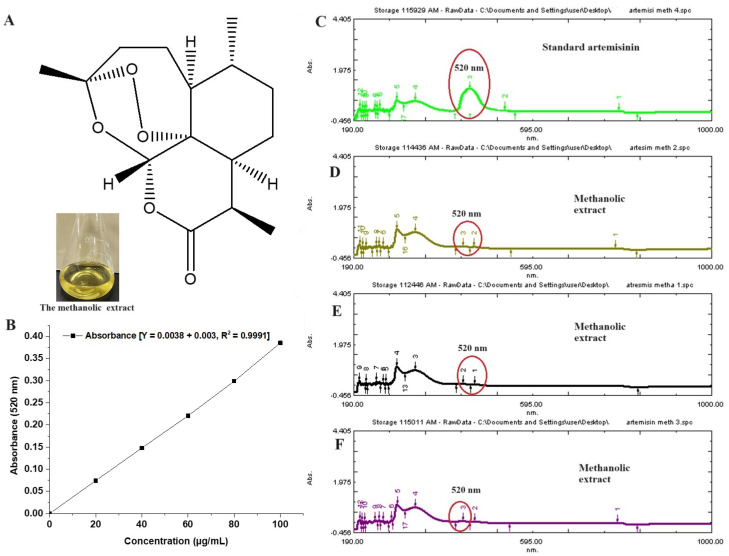
(**A**) Chemical structure of artemisinin, (**B**) standard calibration curve of artemisinin in methanol, (**C**) UV vis spectroscopy scanning (spectrum) of pure artemisinin, exhibiting characteristic maxima at 520 nm, (**D**–**F**) three replicated scans of methanolic extract of *Artemisia pallens* analyzed at different time points (0, 12, and 24 h), exhibiting the stability of the sample. Encircled area indicates characteristic peak at 520 nm. In the extract, (**D**–**F**) show minor peak as compared to standard pure standard artemisinin (200 µg/mL).

The plant extract is enriched with multiple phytoconstituents to provide several pharmacological benefits. Jha et al. reported about 19 constituents from its oil using a gas chromatography technique [32]. The content of artemisinin varies depending upon the species of artemisia. It was reported as 0.1–0.2%, obtained from leaves and flowers of *Artemisia pallens* [33]. Figure 2A shows the chemical structure of artemisinin, whereas Figure 2B illustrates the standard calibration curve of artemisinin over the concentration range of 20–100 µg/mL, with a correlation regression r^2^ value of 0.999. Figure 2C represents a UV spectral scanning of a standard artemisinin solution (methanol), which showed a marked characteristic peak at 520 nm [18]. The extract is rich with multiple flavonoids, essential oil, triterpenes (sesquiterpenes, methyl cinnamate, ethyl cinnamate, linalool, geranyl acetate, and others), glycosides, phenols, polyphenols, alkaloids, fatty acids, saponins, and hormones. However, the innate antimalarial property is due to the potential artemisinin content. Therefore, it was imperative to estimate its content in the extract. Figure 2D–F show relative peaks of artemisinin (red encircled peak) in the extract. The content of artemisin is relatively low, as compared to other constituents. Therefore, the red encircled peak is less intense as compared to others in the spectrum. The estimated amount of artemisin from the extract was 0.28 % per dry weight of *Artemisia pallens,* which is in good agreement with the published report [18].

### 3.2. HPLC Technique

The technique was adopted to support the UV vis spectrophotometer-based assay. The method is relatively sensitive, accurate, reliable, precise, and reproducible for estimating even the trace content of the drug from a biological sample (extract). Therefore, the developed HPLC method provided the representative chromatograms for the standard artemisinin and the extract containing artemisinin [19]. The representative chromatograms for the standard and the extract-based artemisinin are portrayed in Supplementary Figures S1 and S2, respectively. Notably, a remarkable peak was obtained at a retention time of 5.2 min for the standard artemisinin, whereas the extract exhibited the characteristic peak at an approximate retention time of 5.1 min followed by a minor peak at 10.03 min. Chiang et al. reported a similar pattern of chromatograms with characteristics peaks near the retention time points of 7.5 and 7.7 min for the standard artemisinin and the extract-based artemisinin (from *A. anuua*), respectively [19]. A slight variation in the retention time points may be correlated to the slight change in the mobile phase composition, pH, and experimental condition. Other factors may also be taken into consideration. Notably, artemisinin was identified and estimated by comparing the retention time point of the characteristic chromatographic peak generated from the extract with the injected standard artemisinin peak (Supplementary Figures S1 and S2). The estimated content of artemisinin from the extract was found to be 0.431% per dry weight of *Artemisia pallens,* which is within the reported range (0.07–0.45% *w*/*w*) of all artemisia species [18]. The estimated value is approximately double the value obtained through the UV vis spectrophotometer technique. Thus, the HPLC technique was accurate and precise in estimating the trace content of artemisinin from the extract.

### 3.3. Acute Oral Toxicity

The drug is widely used as a traditional medicine for multiple benefits (antioxidant, analgesic, antidiabetic, and anti-inflammatory), with no literature evidence substantiating its safety in animals and humans [34]. Therefore, it is imperative to investigate the maximum and minimum safe oral doses in the explored animal models (mice). The objective was to investigate the safety of the methanolic extract by observing behavioral changes or mortality after acute administration in mice. At 2000 mg/kg p.o. dose, a methanolic extract of *A. pallens* Wall (MEAP) did not cause any behavioral abnormalities or mortality in mice after oral delivery. There was no incidence of general behavioral adverse effects at the explored dose, which may be attributed to the protective nature of the extract [34]. This may be correlated to the optimal dose selected in the study. In the literature, the LD_50_ (lethal dose for 50% mortality) value after acute oral dose administration of a similar genus (*A. afra*) was 8.96 g/kg in mice, which is quite higher than the studied dose [35]. Honmore et al. reported various oral doses of the extract in a rat model during acute toxicity assessment over 14 days. The authors explored 100–700 mg/kg oral doses without any abnormalities and mortality except a fluctuation in biochemical biomarkers [34]. However, the authors did not investigate the radiological analysis of joints followed by histopathological inspection. Therefore, we aimed to address these insights in rat models to understand clear pathological and pharmacological aspects of the extract at the explored concentrations. Thus, the MEAP concentrations chosen for further investigation were 100 (group IV), 200 (group V), and 400 (group VI) mg/kg for oral administration.

### 3.4. Anti-Arthritic Activity

#### 3.4.1. Effect of MEAP on the Body Weight

**Figure 3:**
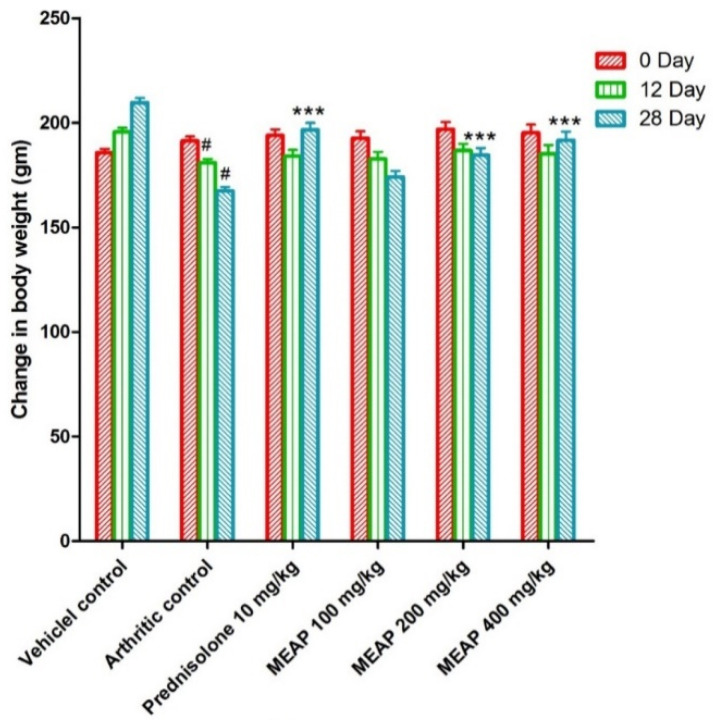
Effect of MEAP on the body weight. Data are expressed as mean ± S.E.M.; *n* = 6 rats per group. Two-way ANOVA followed by Bonferroni’s post hoc test when compared with arthritic control group *** *p* < 0.001, when compared to vehicle control # *p* < 0.001.

The animals were treated as per scheduled doses (100, 200, and 400 mg/kg) of MEAP (Figure 3). Figure 3 shows the routine observed body weight changes in the control and the treated groups on day 0, day 12, and day 28. In general, animals are sensitive to treatment with drugs such as prednisolone and similar drugs in terms of body weight. Therefore, it was mandatory to observe the body weight of the treated animals as compared to the control. The body weight of the arthritic control group (group II) was found to be considerably decreased as compared to group I. Group III was treated with prednisolone (10 mg/kg) and the body weight started to increase significantly from day 20 as a side effect, as compared to group II, due to the steroidal nature of the drug (reduced metabolic rate) [36]. Notably, group IV (treated with 100 mg/K of MEAP) did not show a significant increase in body weight from day 24 as compared to the arthritic control group (group II). Group V treated with MEAP (200 mg/kg) demonstrated a profound increase in body weight on day 28. A similar observation was made with group VI, receiving the highest dose of MEAP (400 mg/kg).

#### 3.4.2. Effect of MEAP on Paw Volume

**Figure 4:**
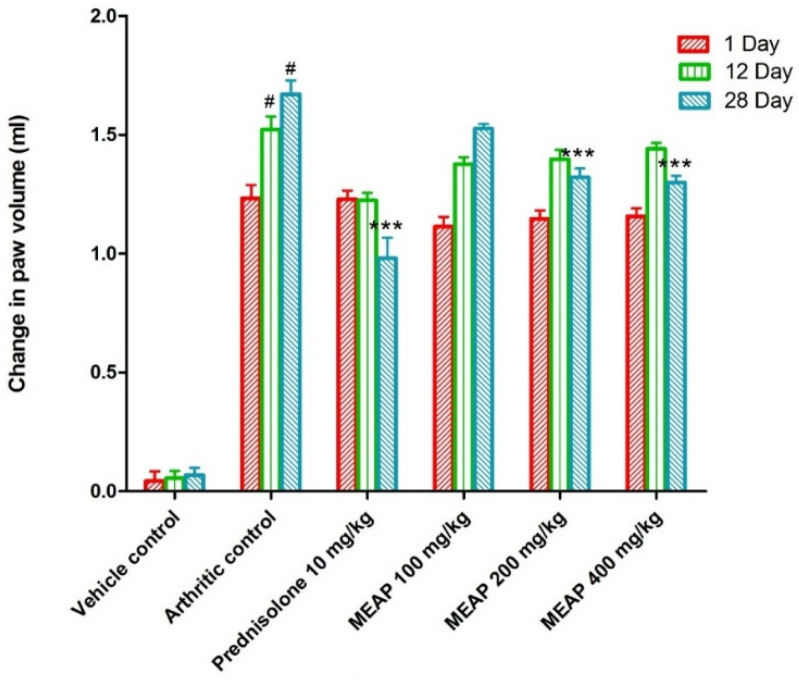
Effect of MEAP on paw volume. Data are expressed as mean ± S.E.M.; *n* = 6 rats per group. Two-way ANOVA followed by Bonferroni’s post hoc test when compared with arthritic control group *** *p* < 0.001, when compared to Vehicle Control # *p* < 0.001.

Comparing the vehicle control group (group I) to the arthritic control group (group II), a significant increase in paw volume was observed. Rats treated with prednisolone (10 mg/kg) on days 1, 12, and 28 exhibited a considerable (*p* < 0.001) reduction in the elevation of their paw volume as compared to the control group (rats without arthritis). Paw volume was not completely lowered by MEAP at 100 mg/kg as compared to the arthritic control group on day 1. However, the paw volume was strongly suppressed as compared to the arthritic control group on the 12th and 28th days at the same dose (*p* < 0.001) (Figure 4). Two doses (200 and 400 mg/kg p.o.) demonstrated a slight variation in paw volume on days 12 and 28, as shown in Figure 4. The highest dose (400 mg/kg) resulted in a substantial reduction in paw volume, which may be attributed to the healing tissue shrinkage. Our findings were in good agreement with the published report, wherein dose-dependent incomplete paw volume reduction (68.85 %) was observed within 3 h at 100 mg/kg of an oral dose of the methanolic extract in a rat model [37]. In the study, there was no dose-dependent ameliorative effect of the extract on days 12 and 28 at 200 and 400 mg/kg doses. Thus, the lower lose (100 mg/kg) could be promising to heal arthritis with high patient compliance (oral administration) and safety if formulated in a suitable dosage form.

#### 3.4.3. Effect of MEAP on the Joint Diameter

**Figure 5:**
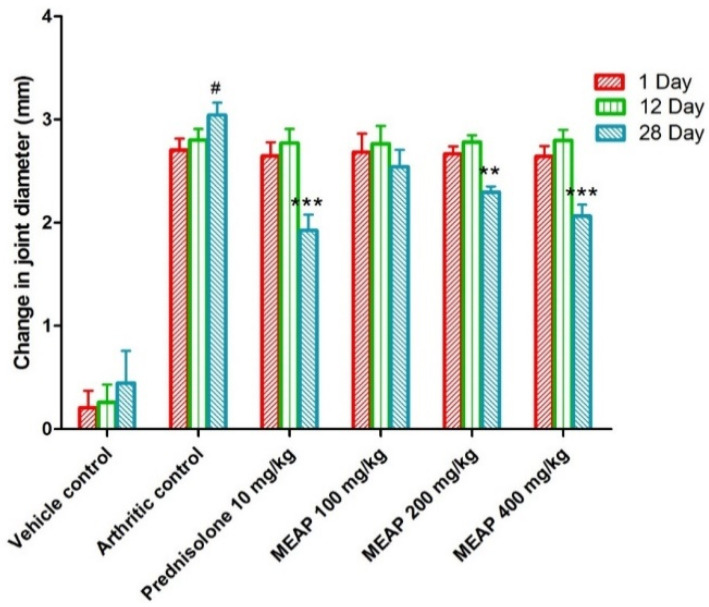
Effect of MEAP on joint diameter. Data are expressed as mean ± S.E.M.; *n* = 6 rats per group. Two-way ANOVA followed by Bonferroni’s post hoc test when compared with arthritic control group ** *p* < 0.01, *** *p* < 0.001, when compared to vehicle control # *p* < 0.001.

The joint diameter of the arthritic control group (group II) was significantly decreased as compared to the vehicle control group I. Prednisolone (10 mg/kg) considerably (*p* < 0.001) reduced the joint diameter on day 28. Observing Figure 5, it is apparent that there is no dose-dependent reduction in the diameter on days 1 and 12. However, there was a progressive reduction (*p* < 0.001) in the diameter on day 28 with an increase in dose. This may be attributed to the pathological healing process and immunological pathway’s functional, with delayed response after oral administration. To execute a pharmacological response, a drug needs to follow a certain pathway of biological healing, with a combination of physiological and drug-related factors (formulation attributes and physicochemical attributes of the extract). Therefore, the extract might have exhibited a significant dose-dependent effect on day 28 as compared to days 1 and 12.

#### 3.4.4. Effect of MEAP on Mechanical Hyperalgesia

**Figure 6:**
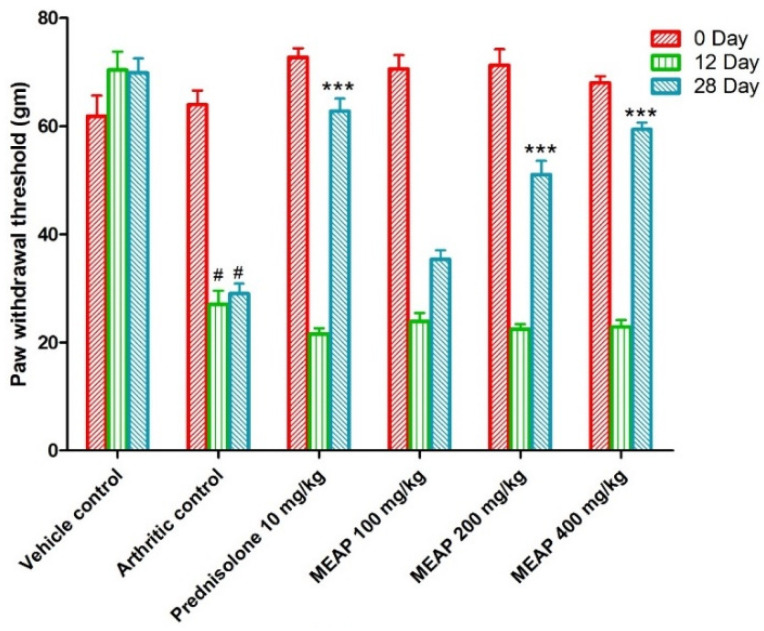
Effect of MEAP on mechanical hyperalgesia. Data are expressed as mean ± S.E.M.; *n* = 6 rats per group. Two-way ANOVA followed by Bonferroni’s post hoc test when compared with arthritic control group *** *p* < 0.001, when compared to vehicle control # *p* < 0.001.

The mechanical withdrawal threshold in the arthritic control group was significantly lower than vehicle control group I, reaching its lowest point on day 12. On days 0, 12, and 28, the mechanical withdrawal threshold of the animal treated with prednisolone (10 mg/kg) significantly (*p* < 0.001) increased as compared to the arthritic control. The MEAP 400 mg/kg (*p* < 0.001) and MEAP 200 mg/kg (*p* < 0.05) treatment groups exhibited significantly greater mechanical withdrawal threshold on day 28 as compared to the arthritic control group. Nevertheless, MEAP at the dose of 100 mg/kg did not considerably change the mechanical withdrawal threshold in relation to the arthritic control (Figure 6).

#### 3.4.5. Effect of MEAP on Thermal Hyperalgesia

**Figure 7:**
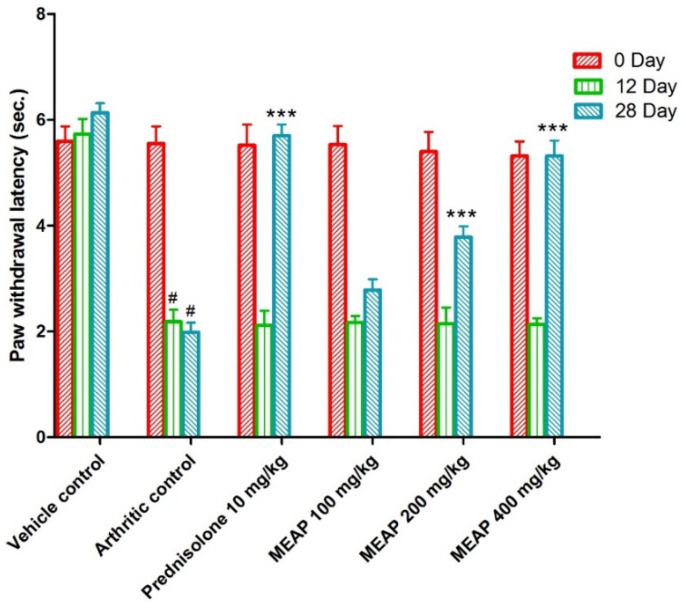
Effect of MEAP on thermal hyperalgesia. Data are expressed as mean ± S.E.M.; *n* = 6 rats per group. Two-way ANOVA followed by Bonferroni’s post hoc test when compared with arthritic control group *** *p* < 0.001, when compared to Vehicle Control # *p* < 0.001.

On comparing the arthritic control against the vehicle control, the paw withdrawal latency (PWL) was considerably decreased. It is important to notice that the paw withdrawal latency of the arthritic control group showed a substantial (*p* < 0.001) increment as compared to the prednisolone-receiving group on days 0, 12, and 28. The lowest selected dose (100 mg/kg) was comparable to the prednisolone, as shown in Figure 7 (on days 0 and 12). Moreover, two subsequent doses (MEAP 400 mg/kg and 200 mg/kg) significantly increased (*p* < 0.001) the PWL on days 12 and 28, respectively, in contrast to the arthritic control. Nevertheless, MEAP (100 mg/kg) had no discernible influence on PWL, as compared to the arthritic control (Figure 7).

#### 3.4.6. Effects of MEAP on Biochemical Parameters

Artemisia species are known to improve biochemical parameters, as reported in various studies. Therefore, it was expected that varied doses of the extract may control the abnormal levels of AST, ALT, and APL in rat models. The result is summarized in Table 1. The positive control (the arthritic control) showed augmented levels of AST, ALT, and ALP in the liver as compared to the vehicle control. Moreover, prednisolone (10 mg/kg) and MEAP at 200 and 400 mg/kg doses significantly reduced AST, ALT, and ALP in comparison to the arthritis control. Reductions in AST, ALP, and ALT levels were found to be dose-dependent. However, the MEAP 100 mg/kg extract dose did not reduce the level of AST, ALT, and ALP to a significant value (Table 1).

#### 3.4.7. Effects of MEAP on the Hematological Parameters

Hemoglobin (Hb) and red blood cell counts were lowered in the arthritic control as compared to the vehicle control, whereas platelet and white blood cell counts were profoundly increased. In contrast to the arthritis control, prednisolone and MEAP treatment at doses of 200 and 400 mg/kg remarkably lowered WBC (white blood cell) and platelet counts, whereas RBC counts and hemoglobin levels were increased. The hematological parameters were not altered by MEAP at 100 mg/kg dose (Table 2).

#### 3.4.8. Effects of MEAP on Antioxidant Parameters

Generally, rats with arthritis showed considerably lower values of liver SOD and GSH, whereas MDA level was found to be high in the blood. However, animals treated with MEAP at doses of 200 and 400 mg/kg, and prednisolone at 10 mg/kg showed considerably higher SOD and GSH values and lower MDA level as compared to the arthritic control group (Table 3). The highest antioxidant potential of *A. pallens* extract is most likely due to its high phenolic content. The extract is rich with phenolic flavonoids (flavonols, flavones, coumarins, phenolic acids, and various miscellaneous compounds) working as antioxidant and reducing properties. Antioxidant phenolic compounds of *A. pallens* can prevent oxidation of lipids and related biomolecules by interrupting the initiation or propagation of oxidation chain reactions. Antioxidant capacity of flavonoids is associated with their redox properties, and it is considered to be inversely related to the lipid peroxidation, cell aging (arthritis), and cancer [38]. Moreover, the anti-arthritis potential of *A. pallens* can be explained based on its innate potential for alleviating inflammation and inducing apoptosis in human RA fibroblast-like synoviocytes, as explored in rats [39]. Honmore et al. reported a similar pattern of findings when a methanolic extract of the drug was administered to rats (pretreatment of the extract at 200 and 400 mg/kg p.o.) [34]. The result is quite convincing and in good agreement with the reported outcomes in the same animal model.

#### 3.4.9. Effects of MEAP on Change in Spleen Weight

There was a significant increase (*p* < 0.001) in the weight of the spleen in the animals with arthritic conditions caused by FCA as compared to the vehicle control. As compared to the arthritis control group, the prednisolone (10 mg/kg) group exhibited a considerable reduction (*p* < 0.05) in the weight of the spleen. The spleen weight of arthritis rats treated with MEAP at doses of 100 mg/kg or 200 mg/kg did not change as expected. However, the dose of 400 mg/kg did not significantly prevent the changes in the spleen weight as compared to the arthritic control group (Table 4).

#### 3.4.10. Radiological Analysis

**Figure 8:**
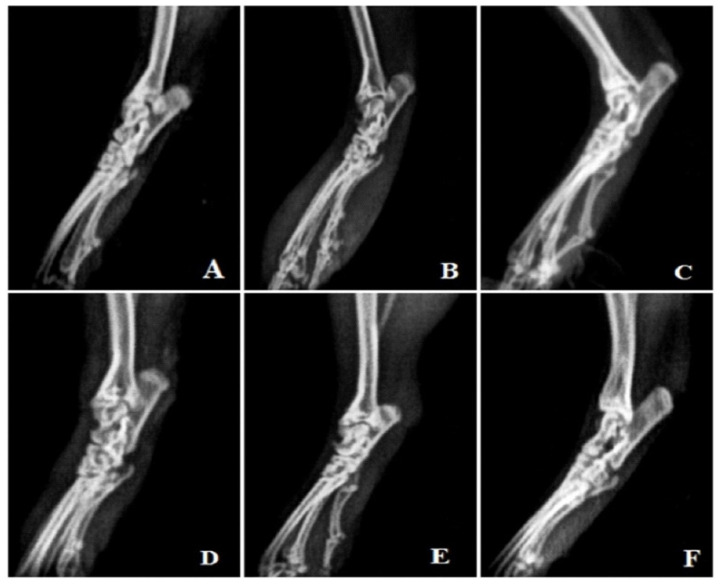
Radiological analysis. (**A**) Vehicle control, (**B**) arthritic control, (**C**) prednisolone 10 mg/kg treatment, (**D**) MEAP 100 mg/kg treatment, (**E**) MEAP 200 mg/kg treatment, and (**F**) MEAP 400 mg/kg treatment.

Rats treated with FCA endured cystic enlargement of the bone, diffuse soft tissue swelling involving the digits, definite joint space narrowing of the intertarsal joints, diffuse demineralization of bone, significant periosteal thickening, and extensive erosions lead to pseudo-widening or narrowing of all joint spaces. As opposed to this, anomalies observed in rats treated with MEAP were primarily limited to the proximal regions of the paws and included periosteal thickening, slight joint space constriction, asymmetric soft tissue swelling, and small erosions (Figure 8).

#### 3.4.11. Histopathological Study of Synovial Joint

**Figure 9:**
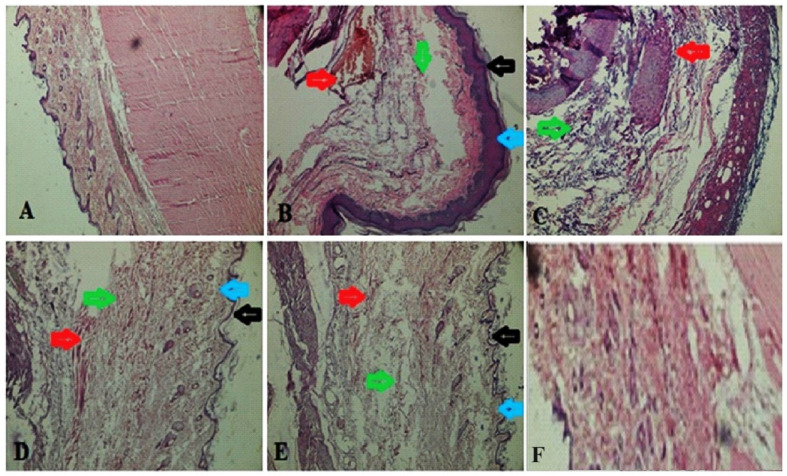
Histopathology of synovial joint. (**A**) Vehicle control; (**B**) arthritic control (blue and black arrows indicate epidermis area showing inflammation, green arrows indicate swelling area filled with edema fluid, and red arrows show inflammation); (**C**) prednisolone (10 mg/kg) treatment, showing green healed area, whereas red arrows indicate removal of fluid; (**D**) MEAP (100 mg/kg) treatment, showing healed epidermis (blue and black arrow), lack of inflammation (green arrow), and recovered swelling (red arrow); (**E**) MEAP at 200 mg/kg treatment elicited recovered swelling, healed area, and treated inflammation; and (**F**) MEAP at 400 mg/kg treatment (completely treated synovial joint).

A histopathological study of the synovial joint of the vehicle control rats revealed that the synovium was still intact (Figure 9). There was no sign of inflammation or an influx of inflammatory cells or cascades. Rats treated with FCA showed pannus development, fibrin deposition, chronic inflammation, deterioration of cartilage, and an inflow of inflammatory cells. Rats receiving prednisone demonstrated notable protection against the degeneration of cartilage, vascular proliferation, thickening of the synovial space, low inflow of inflammatory cells, and the absence of pannus development. Rats treated with 400 mg/kg of MEAP had reduced cartilage loss, thick synovial spaces, increased vascular growth, decreased inflammatory cell infiltration, and no pannus development. After the administration of MEAP (200 mg/kg), rats exhibited mild cartilage loss, thickening of the synovial space, and a small infiltration of inflammatory cells. Rats receiving MEAP at 100 mg/kg oral dose exhibited limited inflammation and a small inflow of inflammatory cells in the synovium, along with signs of a disrupted synovial lining or the development of pannus, as revealed in radiological and histological biopsy studies.

## 4. Discussion

Rats with FCA-induced arthritis are a frequently employed animal model for preclinical investigation of NSAID and disease-modifying anti-rheumatic drugs (DMARDs). It is recommended as the most practical approach for researching medications that affect arthritic patients. It is frequently utilized to investigate the mode of action and preventative measures of several DMARDs [40,41]. Like human rheumatoid arthritis, the development of rat adjuvant-induced arthritis can be classified into three stages. These stages include (1) the induction phase, in which there is no indication of synovitis; (2) early synovitis; and (3) late synovitis, in which there is gradual joint deterioration [41]. Subcutaneous or intradermal injection of FCA results in rheumatoid arthritis-like chronic relapsing arthritis. In this study, macrophages and immune-mediated chronic synovial inflammation are key components. Following activation, they can synthesize cytokines, including TNF-α, and interleukin (IL)-1 as well as mediators like PGE2. Consequently, a range of enzymes that cause cartilage and bone degradation, were produced as a result of their synthetic products [42,43].

The current investigation has shown that oral MEAP treatment substantially reduced the incidence of FCA-induced arthritis in the Wistar rats. The body weight of arthritic control animals was significantly decreased, which may be a subsequent symptom of arthritis or result from the decreased ability of the rat gut to absorb glucose and leucine in an arthritic state [44]. A peak inflammatory response involving fluid exudation, neutrophil infiltration, and mast cell activity was stimulated by FCA administration. This was followed by a gradual regression, and because of the antigenicity of *Mycobacterium* and oil-based adjuvant, the joint swelling persisted until day 28 [45]. The increase in joint diameter and paw volume reduction by MEAP treatment were dose-dependent. The potential of MEAP to impede cellular inflow and vascular permeability may account for the reduction in paw volume and joint diameter. Peripheral pain, hyperalgesia, and functional impairment are the consequences of FCA-induced arthritis. Neurons in the injected paw become allodynia- and hyperalgesia-sensitive. According to several reports, the hyperalgesia associated with arthritis may be influenced by prostaglandin production [46]. The animals of the control group could only bear minimal weight due to their severe arthritic conditions, whereas the MEAP-treated animals (400 and 200 mg/kg) could significantly tolerate bearing their own body weight. According to the results of this study, plantar regions of rats responded less painfully to MEAP at 400 and 200 mg/kg. A key parameter for assessing the disease state in rats with arthritic conditions brought on by FCA is thermal hyperalgesia. The thermoreceptors in the injected paw of arthritic rats are triggered at a lower threshold than in the non-injected paw or in a normal animal. Consequently, the injected PWL in the group treated with MEAP at all three doses exhibited a prolonged latency period as compared to the arthritic control animals.

There was a noticeable enhancement in the weight of the spleen in animals with arthritis. A stimulatory effect on the immune system is indicated by the increase in the spleen weight. In contrast to the arthritis control group, MEAP at all three dose levels demonstrated an insignificant decrease in spleen weight. However, rats who underwent prednisolone treatment demonstrated a considerable (*p* < 0.05) reduction in spleen weight. Stimulation of the immune system could be responsible for a decrease in spleen weight. In this study, arthritic rats showed decreased levels of Hb (RBCs) and increased counts of WBCs and platelets. Moreover, anemia is considered one of the most common extracellular signs of RA [47]. Reduced Hb and RBC counts in arthritic rats suggested that anemia may be a problem in these animals [48]. The primary cause of the reduction in iron levels in the plasma may be due to iron buildup in the synovial tissue and reticuloendothelial system, which prevents the bone marrow from responding to anemia [49]. The immune system being stimulated in response to harmful microorganism invasion is one possible explanation for the proliferation of WBCs and platelets [50].

Assessment of AST, ALT, and ALP serum levels provides a good and straightforward method for determining the efficacy of the target drug to reduce arthritis. According to the reports, the inflammatory process depends upon the production of biochemical mediators that are biologically active, such as bradykinins, serum AST, and ALT. Rats with adjuvant-induced arthritis may have higher serum ALP levels because of an enhancement in the liver and bone fraction. Furthermore, this implies a localized loss of bone in the form of bone erosion and periarticular osteopenia, even though the enzyme is discharged into the circulation during the process of bone resorption and production [51]. In the current study, rats receiving FCA injections had significantly higher AST, ALT, and ALP serum levels. MEAP at doses of 200 mg/kg and 400 mg/kg showed a considerable decrease in AST, ALT, and ALP serum level. However, AST, ALT, and ALP values are not affected at 100 mg/kg as compared to the arthritis control. The possibility of a drop in organ protection mechanisms and bone loss, which may be caused by a decrease in the release of chemical mediators related to the inflammatory process, is revealed by the reduced enzyme level in adjuvant-induced arthritis caused by MEAP treatment. Essentially, reactive oxygen species (ROS) are considered to be the contributory cause of oxidative stress, and these exhibit a well-established role in arthritis because they function as mediators of tissue destruction [52,53]. This causes circulating immune cells to enter the inflammatory tissue upon activation [52,53]. These cells release ROS and pro-inflammatory cytokines into the surrounding milieu [54,55,56]. The endogenous SOD and GSH are considered to be the natural scavengers of ROS, and these significantly prevent cartilage deterioration brought on by ROS. However, a breakdown in this process during an exacerbation of the cellular response to arthritis encouraged the damage of cartilage and bone, essentially induced by ROS [14,55,56,57,58]. MEAP likely competed with SOD for free radical scavenging and limited the loss of glutathione content and helped to maintain the integrity of cellular membranes. Lipid peroxidation is thought to be a key mechanism of the damage that occurs with RA [58]. Here, arthritic rats had higher levels of MDA. Following MEAP treatment, the high level of MDA was considerably decreased. By preventing the production of free radicals, MEAP (200 and 400 mg/kg) mitigated inflammation.

Radiographic study is a useful diagnostic tool that can be used to assess disease severity. Additionally, soft tissue swelling is the first radiographic indicator of arthritis with notable alterations. Bone erosions and joint space constriction are not apparent until the disease is in its late stages. The FCA-induced arthritic rat (group II) showed swelling in the soft tissue and narrowing down of the inter-articular spaces, indicating the bone loss in the arthritic conditions. The conventional prednisolone did not cause any bone destruction or joint swelling. Significant protection against bone deterioration was demonstrated by MEAP (200 and 400 mg/kg)-treated groups, which displayed minimal soft tissue edema followed by narrowing of the the interarticular spaces. In histological investigations of joints, joint deterioration was noted as a result of polymorphonuclear leukocytes, lymphocytes, macrophages, and monocytes migrating into the synovial fluid, as well as connective tissue necrosis. All these processes produce cytokines that cause inflammation. To preserve joints, it may be advantageous to pharmacologically decrease leukocyte migration, connective tissue growth, necrosis, and accumulation in arthritis [21]. In this work, MEAP has demonstrated a protective role by lowering necrosis, connective tissue proliferation, and leukocyte migration. The flavonoids are reported to inhibit chemokinesis of leukocytes and favor dose-dependent healing [58].

## 5. Future Perspectives

The drug is well explored for multiple therapeutic benefits due to several phytoconstituents active against several diseases, especially arthritis. However, several research gaps still exist due to limited investigations on the drug to treat arthritis, inflammatory diseases, and diabetes. These therapeutic benefits are associated with the high content of flavonoids and terpenes obtained from leaves and oils, respectively. The drug can be further explored for anticancer potential as a standalone treatment or in combination with active biochemicals present in the methanolic extract of the aqueous extract. No information is available about the use of methanolic extract in the treatment of infectious disease such as *mycobacterium* and fungal infections. The scarcity of in vivo data suggests that substantial animal studies are still required to support clinical studies so far.

## 6. Conclusions

Outcomes of the investigation showed that *A. pallens* possessed therapeutic advantages in the treatment of arthritis. Based on the experimental evidence provided, it appears that *A. pallens* works through the anti-inflammatory and antioxidant pathways of biological mechanisms. These results were observed in FCA-induced arthritis rats, whereas treatment with *A. pallens* improved the pathological conditions, such as increased body weight, mechanical threshold, and thermal hyperalgesia, with an inhibitory effect on paw volume, joint diameter, and improved hematological, biochemical, and oxidative parameters. Moreover, the pathological condition was ameliorated by decreased joint space narrowing, swelling of soft tissue, erosion of joint cartilage, and cell infiltration in arthritic control group. The study supported the conclusion that MEAP at 200 and 400 mg/kg doses significantly inhibited the development and progression of arthritis. Summarily, findings of the study are consistent with the hypothesis that *A. pallens* possesses promising analgesic, anti-inflammatory, anti-arthritic effects. Moreover, additional research is still required to glean in vivo data for long-term toxicity assessment in preclinical and clinical setups. Hence, more study is required to completely understand the underlying biological pathways and to validate these findings in clinical situations.

## Figures and Tables

**Table 1:** The effect of MEAP on the biochemical parameters.

Treatment Groups	Dose mg/kg	AST (U/L)	ALT (U/L)	ALP (U/L)
Vehicle control	-	38.12 ± 1.14	43.56 ± 2.46	74.91 ± 2.82
Arthritic control	-	134.60 ± 3.24 ^#^	176.70 ± 1.79 ^#^	442.10 ± 14.02 ^#^
Prednisolone	10	54.54 ± 2.03 ***	55.02 ± 2.04 ***	158.80 ± 8.37 ***
MEAP	100	123.70 ± 3.85	169.10 ± 2.88	421.10 ± 8.75
MEAP	200	118.70 ± 4.20 **	162.00 ± 2.398 **	403.40 ± 10.99 *
MEAP	400	76.89± 2.38 ***	119.70 ± 2.814 ***	395.30 ± 10.16 **

Note: “*” and “**” indicate significance level at *p* < 0.05 and *p* < 0.001, respectively, as compared to the control prednisolone ***. # indicates positive arthritis-induced control group.

**Table 2:** The effect of MEAP on the hematological parameters.

Treatment Groups	Dose mg/kg	Hb (gm/100 mL)	WBC (Thousands/µL)	RBC (Million/µL)	Platelet (Lacks/µL)
Vehicle control	-	14.19 ± 0.27	7.53 ± 0.21	6.87 ± 0.11	9.60 ± 0.56
Arthritic control	-	8.70 ± 0.17 ^#^	15.09 ± 0.27 ^#^	3.66 ± 0.16 ^#^	18.09 ± 0.37 ^#^
Prednisolone	10	13.09 ± 0.36 ***	12.34 ± 0.25 ***	5.69 ± 0.14 ***	11.24 ± 0.29 ***
MEAP	100	9.10 ± 0.20	14.78 ± 0.22	3.79 ± 0.18	17.72 ± 0.20
MEAP	200	9.68 ± 0.23 *	14.00 ± 0.28 *	4.84 ± 0.32 *	16.17 ± 0.60 *
MEAP	400	10.15 ± 0.29 **	13.75 ± 0.33 **	5.18 ± 0.57 **	15.52 ± 0.51 **

Note: “*” and “**” indicate significance level at *p* < 0.05 and *p* < 0.001, respectively, as compared to the control prednisolone ***. # indicates positive arthritis induced in the control group.

**Table 3:** Effect of MEAP on antioxidant parameters.

Treatment Group	Dose mg/kg	SOD (mU/mg Protein)	GSH (µmole/mg Protein)	MDA (nmole MDA/mg Protein)
Vehicle control	-	4.41 ± 0.05	69.56 ± 1.03	1.98 ± 0.05
Arthritic control	-	2.43 ± 0.05 ^#^	43.29 ± 0.90 ^#^	3.42 ± 0.04 ^#^
Prednisolone	10	3.37 ± 0.14 ***	61.26 ± 1.53 ***	2.92 ± 0.08 ***
MEAP	100	2.48 ± 0.04	43.59 ± 1.57	3.29 ± 0.06
MEAP	200	2.86 ± 0.08 **	49.62 ± 1.13 **	3.12 ± 0.09 **
MEAP	400	3.15 ± 0.075 ***	50.66 ± 0.84 ***	3.04 ± 0.05 ***

Note: “**” indicate significance level at *p* < 0.001, as compared to the control prednisolone ***. # indicates positive arthritis-induced control group.

**Table 4:** Effect of MEAP on change in spleen weight.

Treatment Group	Dose (mg/kg)	Spleen Weight (g)
Vehicle control	-	1.00 ± 0.04
Arthritic control	-	1.42 ± 0.06 ^#^
Prednisolone	10	1.82 ± 0.07 *
MEAP	100	1.39 ± 0.04
MEAP	200	1.27 ± 0.06
MEAP	400	1.23 ± 0.05

Note: “*” indicates significance level at *p* < 0.001, whereas # indicates positive arthritis-induced control group.

## Data Availability

The data will be provided on relevant request.

## Supplementary Materials

The following supporting information can be downloaded at: https://www.mdpi.com/article/10.3390/diseases12100230/s1, Figures S1 and S2: HPLC method based estimated artemisinin.
