# Supplementary material for: Artemisia pallens W. Attenuates Inflammation and Oxidative Stress in Freund’s Complete Adjuvant-Induced Rheumatoid Arthritis in Wistar Rats
Source: Diseases. 2024 Sep 29;12(10):230. doi: 10.3390/diseases12100230 (PMC11508142; doi:10.3390/diseases12100230)
Supplement: Supplementary file 1 [file diseases-12-00230-s001.zip › diseases-3141449-supplementary.pdf]

Supplementary file

HPLC method based estimated artemisinin

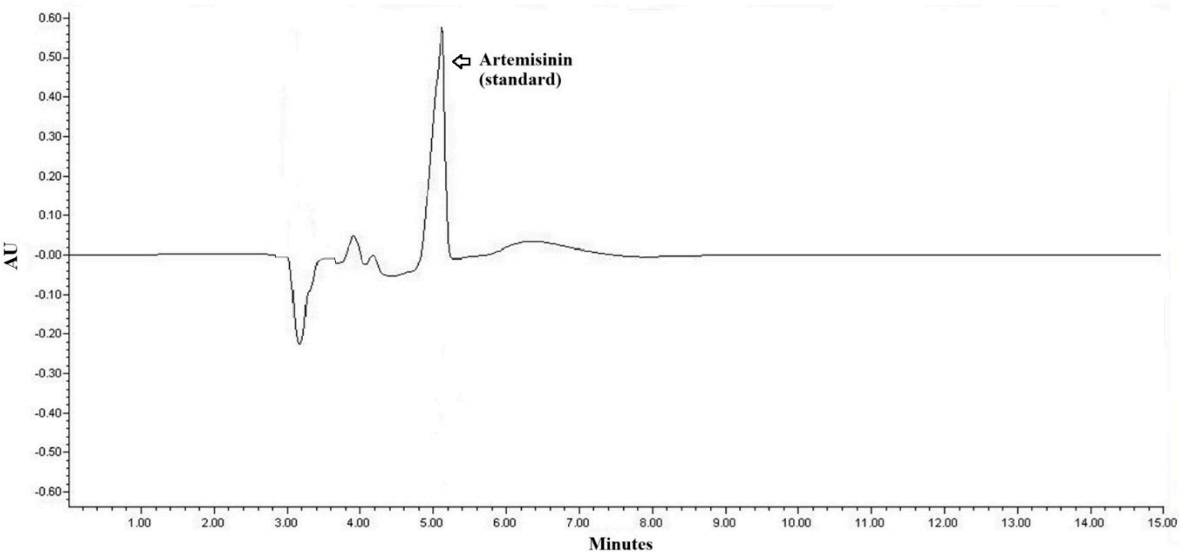

Supplementary figure S1: Representative chromatogram of artemisinin standard in methanol exhibiting characteristic peak at retention time of 5.2 min

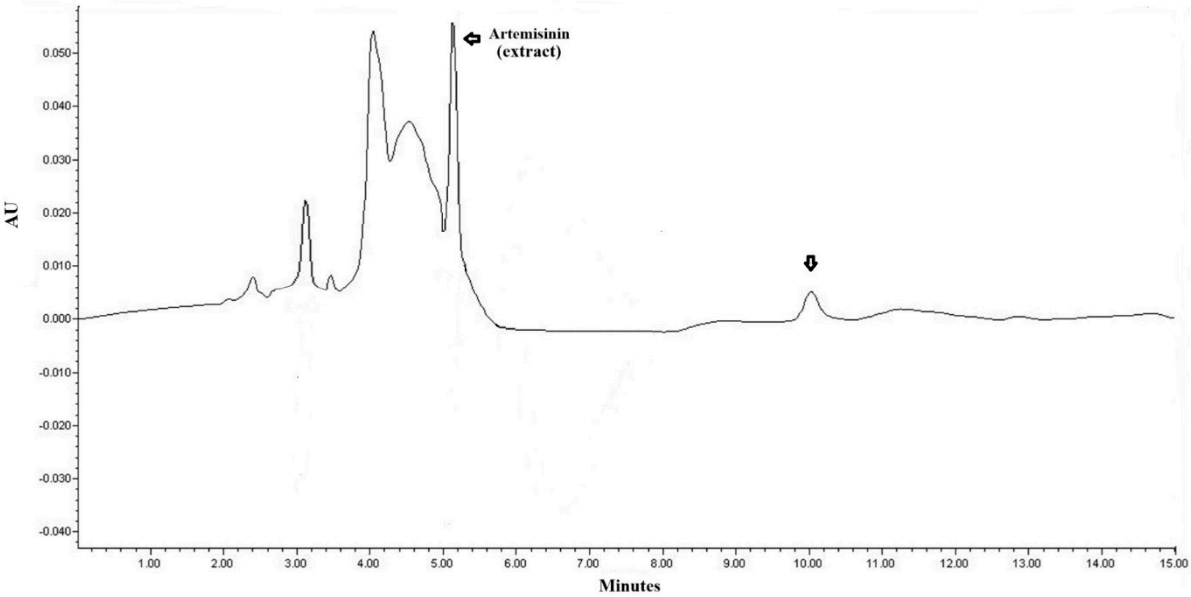

Supplementary figure S2: Representative chromatogram of the extract in methanol exhibiting characteristic peak of artemisinin at retention time of 5.1 min. A minor peak (indicated as a black arrow head) at the retention time of 10.03 min is in good agreement with the published report for the presence of artemisinin in the extract [18].
